# Liposomal Packaging Generates Wnt Protein with In Vivo Biological Activity

**DOI:** 10.1371/journal.pone.0002930

**Published:** 2008-08-13

**Authors:** Nathan T. Morrell, Philipp Leucht, Ludan Zhao, Jae-Beom Kim, Derk ten Berge, Karthik Ponnusamy, A. Lyonel Carre, Henryk Dudek, Marie Zachlederova, Michael McElhaney, Shirley Brunton, Janet Gunzner, Marinella Callow, Paul Polakis, Mike Costa, Xiaoyan M. Zhang, Jill A. Helms, Roel Nusse

**Affiliations:** 1 Division of Plastic and Reconstructive Surgery, Department of Surgery, Stanford University, Stanford, California, United States of America; 2 Department of Developmental Biology, School of Medicine, Stanford University, Stanford, California, United States of America; 3 Curis Inc., Cambridge, Massachusetts, United States of America; 4 Evotec Ltd., Abingdon, Oxon, United Kingdom; 5 Department of Medicinal Chemistry, Genentech Inc., South San Francisco, California, United States of America; 6 Department of Cancer Targets, Genentech Inc., South San Francisco, California, United States of America; Netherlands Cancer Institute, Netherlands

## Abstract

Wnt signals exercise strong cell-biological and regenerative effects of considerable therapeutic value. There are, however, no specific Wnt agonists and no method for *in vivo* delivery of purified Wnt proteins. Wnts contain lipid adducts that are required for activity and we exploited this lipophilicity by packaging purified Wnt3a protein into lipid vesicles. Rather than being encapsulated, Wnts are tethered to the liposomal surface, where they enhance and sustain Wnt signaling *in vitro*. Molecules that effectively antagonize soluble Wnt3a protein but are ineffective against the Wnt3a signal presented by a cell in a paracrine or autocrine manner are also unable to block liposomal Wnt3a activity, suggesting that liposomal packaging mimics the biological state of active Wnts. When delivered subcutaneously, Wnt3a liposomes induce hair follicle neogenesis, demonstrating their robust biological activity in a regenerative context.

## Introduction

Wnt signals are implicated in the self-renewal and proliferation of stem cells from a variety of adult tissues [1]–[3] but despite numerous large-scale screenings [4]–[7], no specific small molecule Wnt agonists have been identified. As a consequence, the therapeutic application of Wnt proteins - of which there are potentially many [8]–[10] - has been severely hampered. We exploited a unique and essential feature of Wnt proteins, their covalent modification by several lipids, to package the purified proteins in a manner that both stabilizes and enhances their *in vivo* biological activity. Palmitoylation is essential for Wnt activity [1], [11]; therefore we reasoned that liposomes might serve as an ideal delivery vehicle for such a hydrophobic molecule.

Liposomes are spherical nanovesicles consisting of an aqueous core enclosed in one or more phospholipid layers (reviewed in [12]). Initially, liposomes were developed in an attempt to improve the pharmacokinetics and tissue distribution of chemotherapeutic agents [13]–[15]. Consequently, bioengineers and chemical engineers have invested considerable time and effort into manufacturing liposomes that retain the drug or molecule of interest and prevent its degradation. Such preparations would ideally effectively evade detection by the reticuloendothelial system; could be targeted to the tissue of interest; and could be induced to release the drug/molecule when required. For example, the addition of polyethylene glycol (PEG) can prolong the circulatory half-life of liposomes, perhaps acting through steric hindrance ([14]–[16]; reviewed in [17]).

At present, the primary application for liposome technology is for the treatment of cancers (reviewed in [14], [15], [18]). In this clinical scenario, the objective is to deliver cytotoxic drugs to a tumor whilst simultaneously preserving cell viability in the rest of the body. Our objective differed slightly: we wanted to develop a method to deliver Wnts to a tissue that simultaneously preserved biological activity and restricted diffusion of the protein.

## Materials and Methods

### Purification of Wnt3a liposomes

Mouse Wnt3a protein was purified as described [1], without the heparin purification step. The product, containing approximately equal amounts of Wnt3a and bovine serum albumin, was concentrated further to 250 ng/µl in PBS with 1% CHAPS.

### Generating Wnt3a liposomes

Many different lipid compositions were attempted. In all cases, 14 µmol of lipid were added; when multiple lipids were used, they were added in a 90∶10∶4 molar ratio as indicated. 1,2-Dipalmitoyl-*sn*-Glycero-3-Phosphocholine (DPPC)(cat#: 850355C), 1-Myristoyl-2-Palmitoyl-*sn*-Glycero-3-Phosphocholine (MPPC)(cat#: 850445C), 1,2-Distearoyl-*sn*-Glycero-3-Phosphoethanolamine-N-[PDP(Polyethylene-Glycol)2000] (DSPE-PEG_2000_)(cat#:880129C), 1,2-Dimyristoyl-*sn*-Glycero-3-Phosphocholine (DMPC)(cat#:850345C), and 1-Palmitoyl-2-Oleoyl-*sn*-Glycero-3-Phosphocholine (POPC)(cat#:850457C) were obtained from Avanti Polar Lipids, Inc. (Alabaster, AL).

Unless otherwise indicated, 14 µmol of DMPC in chloroform were dried to a thin film in a 10 ml round bottom flask using nitrogen gas and were further evaporated in a vacuum overnight. Purified Wnt3a in 1% CHAPS in 1× PBS was then diluted in 1× PBS to a total concentration of 1–1.3 µg/ml. This solution was then added to the 10 ml flask and vortexed vigorously until the solution was cloudy and there was no lipid visible on the bottom of the flask. The lipid solution was then extruded 40 times through a 100–200 nm polycarbonate membrane in a thermo-barrel extruder held at 30–32°C (Avanti Polar Lipids, Inc).

To separate liposome-associated Wnt3a from free Wnt3a, the extruded solution was spun in an Optima TLX Ultracentrifuge (Beckman Coulter, Fullerton, CA) at 28,000 rpm for a minimum of 30 minutes at 4°C. The supernatant was removed and the lipid pellet re-suspended in 1× DMEM (Mediatech, Inc., Herndon, VA). The liposomes, if not used immediately, were stored at 4°C.

### Cell culture and activity assays for Wnt3a liposomes

Mouse LSL cells were grown at 37°C and 5% CO_2_ in 1× DMEM, 10% FBS, and 1% Penicillin/Streptomycin (Mediatech, Inc., Herndon, VA). The cells were plated in 96-well plates with an initial density of 25,000 cells/well and allowed to recover overnight. The cells were then treated as described and incubated for an additional 17 hours. As the LSL cells constitutively express β-galactosidase and express luciferase in response to Tcf/Lef binding, activity was assessed via the Dual-Light® Combined Reporter Gene Assay System (Applied Biosystems). Relative luciferase units were measured and normalized against β-galactosidase activity. Error bars indicate standard deviation. All assays were done in triplicate. Student's T test was employed to determine statistical significance.

### Determination of effective Wnt3a concentration in Wnt liposomes

Increasing amounts of purified Wnt3a protein were added to LSL cells, grown in 96-well plates, in order to generate an activity gradient. In a parallel set of experiments, LSL cells were exposed to different volumes of Wnt3a liposomes. All experiments were performed in triplicate. Activity of the purified protein was then determined by luciferase activity (as described above) and liposomal Wnt3a activity was plotted on the same graph. From these data we interpolated the concentration of active Wnt3a in a given volume of liposomes.

### Trypsin digestion of Wnt3a liposomes

Exo-liposomal Wnt3a protein was removed by subjecting liposomes to trypsin digestion. Briefly, 75 µl of Wnt3a liposomes were added to 405 µl of 1× DMEM and 20 µl 1× Trypsin solution (Mediatech, Inc., Herndon, VA) for a final mixture containing 4.0% trypsin. Liposomes were then incubated at 37°C for 0, 5, 10, 15, or 20 minutes. The proteolytic reaction was quenched by the addition of 1 ml of 1× DMEM containing 10% FBS. The samples were ultra-centrifuged at 28,000 rpm for 30 min at 4°C; the supernatant was then removed and the lipid pellet re-suspended in 1× DMEM. The samples were then added to LSL cells in triplicate, or prepared for standard Western blot analysis.

### Isolation and characterization of Wnt antagonists

We undertook a small molecule Wnt antagonist high-throughput screen using a mammalian cell–based reporter assay. The reporter plasmid (Super8xTOPflash) was stably introduced into the mouse C3H10T1/2 cell line, and the best 10T1/2 Super8xTOPflash clone gave ∼800 fold up-regulation of luciferase activity after 24 hour incubation with Wnt3a conditioned media.

Under these assay conditions we screened ∼205,000 small molecule compounds at a concentration of 10 µM and identified several potential Wnt antagonists (hereafter referred to as Ant). These antagonists were further evaluated using a luciferase reporter driven by the SV40 large T antigen promoter (for detecting general non-specificity) and a minimal promoter with multimerized Gli binding sites (for detecting activity on the Hedgehog pathway). The primary screen hit for the compound series described in this paper was effective at inhibiting Wnt signaling (IC50∼1–2 µM) but did not function as a Hedgehog inhibitor (IC50>20 µM) and showed no inhibitory effect on SV40 promoter driven luciferase reporter at concentrations up to 30 µM. In the derivative Ant 1.4, either a bromide or a chlorine atom was substituted in the ortho position to enhance activity over the unsubstituted molecule.

### Evaluation of Wnt antagonist activity in autocrine and paracrine cultures

C3H10T1/2 cells were transfected with a Super8xTOPflash luciferase reporter plasmid (Upstate Cell Signaling Solutions). PA-1 cells (ATCC) were transfected with a TOPflash (Upstate Cell Signaling Solutions) luciferase reporter plasmid. Both cell lines were maintained using standard tissue culture protocols.

PA-1 cells exhibit autocrine Wnt signaling [19]; to monitor the ability of antagonists to block autocrine Wnt activity, cells were plated in 96-well plates at 20,000 cells/well in growth medium; 24 hrs later, the cells were changed to fresh growth medium and the antagonists were added in the presence of 20% Wnt3a condition medium, or 0.1 µg/ml purified Wnt3a protein. After 24 hrs, plates were assayed for luciferase activity with the LucLite kit (Packard).

To test the ability of the antagonists to block an exogenous Wnt signal and autocrine Wnt signal simultaneously, PA-1 cells transfected with Super8xTOPflash reporter were plated as described above; 24 hrs later, the cells were changed to fresh growth medium and the antagonists were added in the presence of 20% Wnt3a condition medium, or 0.1 µg/ml purified Wnt3a protein. After 24 hrs, plates were assayed for luciferase activity with the LucLite kit (Packard).

To test the ability of the antagonists to block paracrine Wnt signaling, we co-cultured 10T1/2 Super8xTOPflash reporter cells with L cells stably expressing Wnt 3a protein. In all assays, hFzd8 CRD, an antagonist of Wnt signaling [19], served as a positive control for Wnt inhibition.

### Wnt antagonist activity: liposomal preparation

Wnt antagonist (Ant) 1.4Cl was diluted from 20 mM in DMSO to final concentrations of 2 µM or 20 µM in 1× DMEM. LSL cells were treated with 15 µL of Wnt3a liposomes or 15 µL of purified Wnt3a protein and varying concentrations of Ant 1.4Cl ranging from 0.01 µM to 10 µM and allowed to incubate overnight. The activity assay was conducted as previously mentioned and was conducted in triplicates. Percent inhibition values are relative to non-antagonist treated Wnt3a liposome or free Wnt3a protein activity as appropriate.

### Animal studies

All experiments were performed in accordance with Stanford University Animal Care and Use Committee guidelines. Six three-week old, male CD-1 mice were purchased from Charles River Laboratories, Inc. (Wilmington, MA). Animals were housed in a light- and temperature-controlled environment and given food and water ad libitum.

Before subcutaneous injection of Wnt3a/PBS liposomes, the back of the mice was shaved and cleansed with antiseptic Betadine. Two regions, each 1 cm in diameter, were marked approximately 5 mm lateral from the midline. Mice received subcutaneous injections of either 20 µl Wnt3a liposomes or PBS liposomes every other day. Tissues were harvested after 14 days (7 injections) and fixed in 4% Paraformaldehyde overnight. Samples were processed for paraffin embedding, sectioned at 8 µm thickness and stained with H&E for histological evaluation.

## Results

Our first goal was to determine if liposomal packaging affected the activity of Wnt3a protein. We generated Wnt3a liposomes and compared their activity to purified soluble Wnt3a protein using the LSL dual-reporter cell assay [20]. These cells show robust, dose-dependent expression of luciferase in response to Wnt3a treatment (Fig. S1).

**Figure 1:**
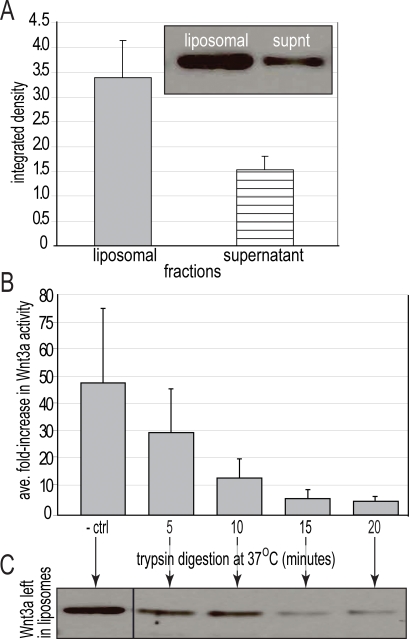
Active Wnt3a protein is associated with the exo-liposomal surface. (A) Western analyses indicated that ∼70% of Wnt3a protein added during synthesis was incorporated into the liposomes; the remaining 30% remained in the supernatant. (B) Trypsin removed liposomal Wnt3a activity as measured in an *in vitro* dual-reporter assay (n = 3, mean+standard deviation). (C) Western analyses demonstrated that ∼20% of Wnt3a protein incorporated into the liposomal preparation still remained following trypsin digestion. This portion (∼20%) was localized to the endo-liposomal surface where it was inactive.

In our initial formulation of Wnt liposomes we first tested the activity of Wnt protein associated with a liposome. The liposomal preparation of Wnt3a was made by combining 1.0 µg/ml of Wnt3a protein with lipids to generate a solution with an estimated concentration of 0.5 µg/ml. Instead of being distributed equivalently in the supernatant and the pellet, however, Western blot analyses indicated that the lipid pellet contained the majority of the Wnt3a protein (Fig. 1A). This lipid-associated Wnt3a exhibited activity using the same luciferase assay (Fig. 1A).

Because of its lipid modifications, we speculated that Wnt3a protein would associate with the lipid bilayer in such a way that Wnt positioned on the exo-liposomal surface would be available for receptor binding but Wnt positioned in the endo-liposomal surface would not. To test this hypothesis we subjected the Wnt3a liposomes to trypsin digestion to remove protein on the exo-liposomal surface. Trypsin digestion completely inactivated the Wnt3a liposomes (Fig. 1B). The ∼20% of the Wnt3a protein remained associated with the liposomal fraction where it did not elicit any activity (Fig. 1C). We therefore conclude that the majority (80%) of Wnt3a is positioned on the exo-liposomal surface where it exhibits biological activity, while a small percentage remains localized to the endo-liposomal environment where it is unavailable for signaling (Fig. 1C).

### Liposomal packaging enhances Wnt3a activity

**Figure 2:**
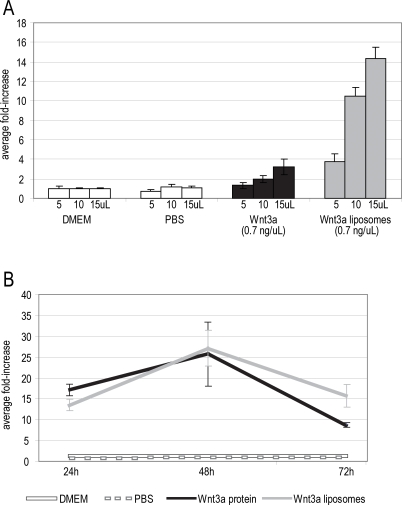
Liposomal packaging of Wnt3a potentiates its biological activity. (A) Equivalent concentrations of Wnt3a and liposomal Wnt3a were tested for their ability to stimulate luciferase activity in LSL cells. DMEM and PBS exhibited baseline activity. Wnt3a protein elicited volume-dependent activity. An equivalent concentration of liposome-packaged Wnt3a exhibited substantially greater activity. (B) Liposomal packaging sustained Wnt3a-dependent activity. The same volume of Wnt3a and Wnt3a liposomes elicited similar levels of activity after 24 h and 48 h *in vitro*. At 72 h, however, liposomal Wnt3a exhibited greater activity than Wnt3a protein.

During fabrication of Wnt3a liposomes, we calculated that ∼55% of the input protein is incorporated in a manner that exhibits biological activity, while the remaining 45% is either lost in the supernatant, or is sequestered in the endo-liposomal space. Does the liposomal presentation of Wnt3a affect its biological activity? We compared the activity of Wnt3a protein against Wnt3a liposomes containing the same concentration of active Wnt3a on the exo-liposomal surface (Fig. 2A). Based on the use of equivalent concentrations of active Wnt3a, we calculate that the liposomal preparation of Wnt3a exhibits a 5-fold increase in biological activity compared to the isolated protein (Fig. 2A).

### Liposomal packaging preserves Wnt3a activity

We also tested whether liposomal packaging preserved the biological activity of Wnt3a over time. TOPflash cells were plated at different cell densities in order to insure that cells were in the exponential growth phase throughout the experiment. Cells were treated with various concentrations of Wnt3a protein, or Wnt3a liposomes that were normalized for having similar activity. Reporter activity was determined at each time point and normalized against cell number. We found that Wnt3a and Wnt3a liposomes exhibited similar biological activity after 24 and 48 h in culture but Wnt3a liposomes showed significantly higher activity after 72 h in comparison to Wnt3a protein (Fig. 2B). Therefore, Wnt3a positioned on the exo-liposomal surface is active, and this packaging scheme potentiates and stabilizes the activity of Wnt3a *in vitro*.

**Figure 3:**
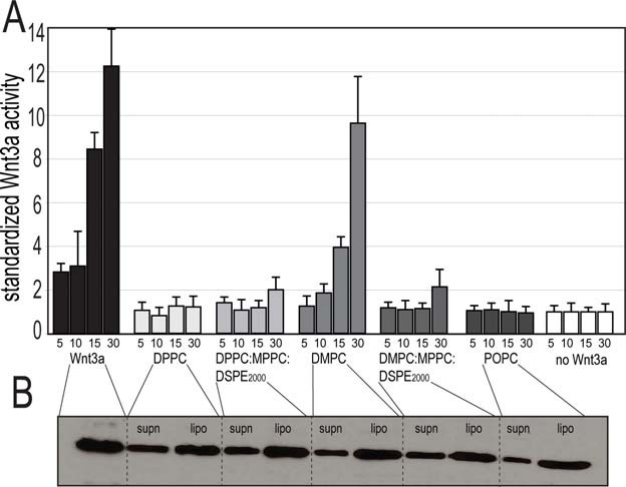
Wnt3a dependent activity is affected by lipid composition. Liposomes were made with various lipid compositions as indicated (n = 3; mean+standard deviation). (A) Different lipid compositions exhibited radically different activities *in vitro*. (B) Western blot analysis showed that these differences in activity were not attributable to variations in total Wnt incorporation into the liposomal membrane.

We examined whether DMPC could be replaced by other lipids with varying carbon side chain lengths (i.e. DPPC has a fourteen carbon backbone whereas DMPC has a twelve carbon backbone). We found that lipid formulations considerably influenced the activity of the liposomes, with DMPC being optimal (Fig. 3), despite the fact that Western analyses indicated that a similar amount of total protein was incorporated into each liposomal preparation (Fig. 3).

### Identification of Wnt antagonists by high through-put screening

**Figure 4:**
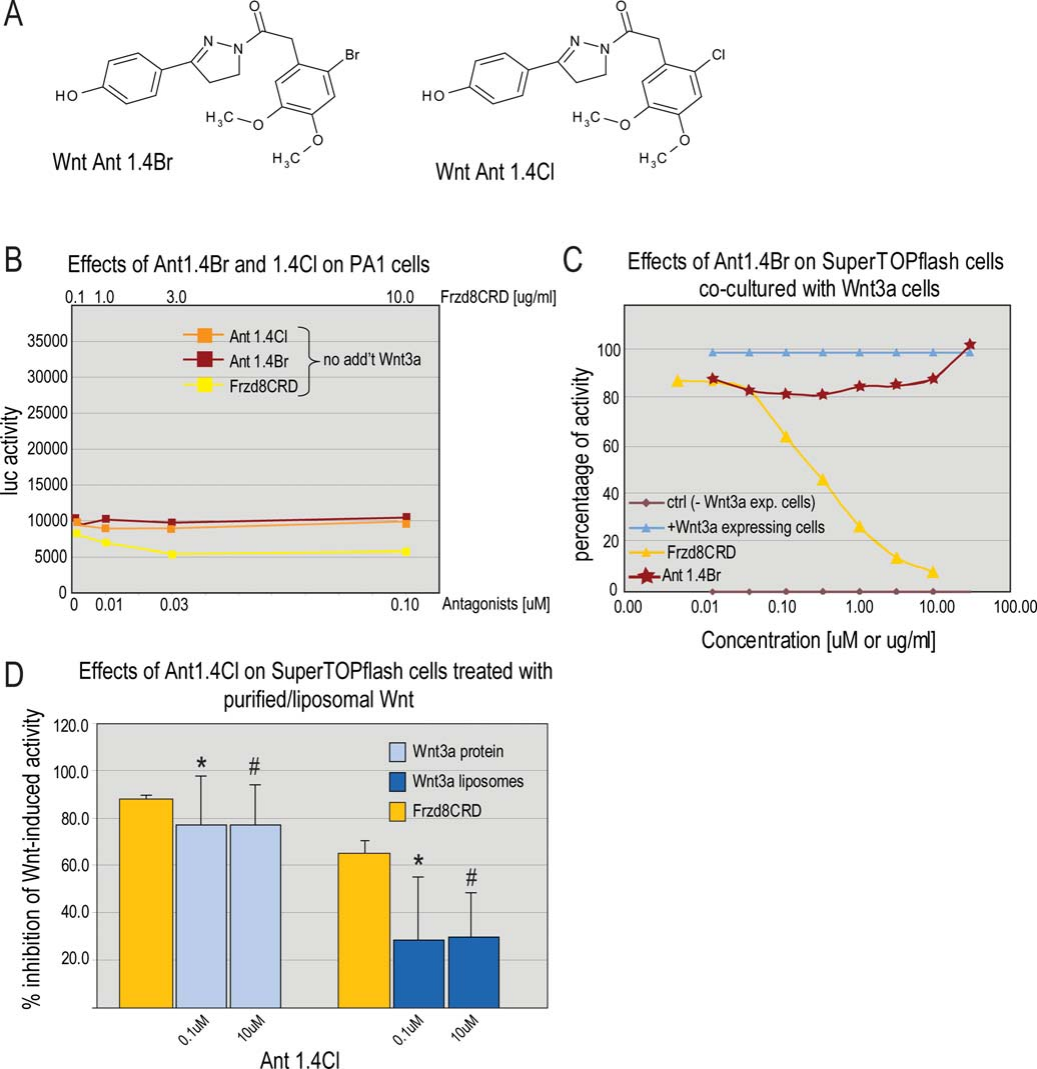
Wnt antagonists are effective against purified Wnt3a but ineffective against autocrine, paracrine, and liposomal Wnt3a. (A) Structure of the 2-bromo 4,5-dimethoxysulfonamide analog (Ant 1.4Br) and 2-chloro 4,5-dimethoxysulfonamide analog (Ant 1.4Cl). (B) PA-1 cells transfected with a SuperTOPflash Wnt reporter were treated with Ant 1.4Br, Ant 1.4Cl, or hFzd8CRD. Frzd8CRD (yellow line) reduced Wnt-dependent luciferase activity. Ant 1.4Br (red) and Ant 1.4Cl (orange) were ineffective at blocking Wnt-induced activity. (C) 10T1/2 cells transfected with a TOPflash Wnt reporter construct were co-cultured with LSL cells secreting Wnt3a. No luciferase activity was detectable in 10T1/2 cells grown alone (control, brown line); luciferase activity was at a maximum when cells were co-cultured with LSL cells secreting Wnt3a (blue line). Fzd8CRD (yellow) inhibited paracrine-induced Wnt reporter activity in a dose-dependent manner, to a maximum of 90%. Ant 1.4Br (red) reduced luciferase activity by less than 20%. (D) LSL cells transfected with a TOPflash Wnt reporter construct were treated with either exogenous Wnt3a or liposomal Wnt3a in the presence of Ant 1.4Cl or hFzd8CRD. Ant 1.4Cl inhibited exogenous Wnt3a-induced reporter activity by 80% but inhibited liposomal Wnt-induced reporter activity by only 30%. hFzd8CRD (yellow) reduced Wnt3a-induced reporter activity by 80% in Wnt3 treated cells and by 70% in liposome treated cells. The symbols * and # reflect statistical significance (Student's T-test, p<0.05).

In parallel series of experiments, we sought to identify molecules that were effective at inhibiting Wnt signaling. In our initial screening we used 10T1/2 cells transfected with the Super8xTOPflash Wnt reporter construct, and treated the cells with soluble Wnt3a. We focused on a molecule that blocked Wnt-induced reporter activity (Fig. 4) and also repressed *Axin2* expression in 10T1/2 cells and *SAX1* and *GAD1* expression in teratocarcinoma cells induced with exogenous Wnt3a (data not shown).

In an effort to improve potency of the antagonists we synthesized over 200 derivatives and tested these derivatives in the 10T1/2 Super8xTOPflash reporter assay. The most potent antagonists had IC50s less than 1 nM, indicating over 1000-fold enhancement in potency by chemical modification (data not shown). Epistasis analysis indicates that this class of small-molecule inhibitors acts upstream of β-catenin at the level of Wnt ligands or receptors (data not shown). Of these, Ant 1.4Br and one of its derivatives, Ant 1.4Cl, were tested further for their ability to inhibit autocrine Wnt signaling (Fig. 4A).

### Antagonists discriminate between an autocrine and an exogenous Wnt signal

The ability of Ant 1.4Br and Ant 1.4Cl to block autocrine Wnt signaling was tested in three separate cell lines (NCCIT, NTera2, and PA-1) all of which were transfected with a luciferase-based Wnt reporter construct. We compared the inhibitory effects of Ant 1.4Br and Ant 1.4Cl against hFzd8CRD. In an autocrine signaling assay, hFzd8CRD repressed luciferase activity by 50% (Fig. 4B). In contrast, Ant 1.4Br and 1.4Cl inhibited luciferase activity by only 10% (Fig. 4B).

Ant1.4Br and 1.4Cl were also ineffective against paracrine Wnt signaling. When cultured alone, 10T1/2 Super8xTOPflash reporter cells showed no luciferase activity but when co-cultured with L cells stably expressing Wnt3a protein, then luciferase activity was increased 25-fold, which was largely inhibited by hFzd8-CRD (Fig. 4C). Ant 1.4Br and its derivatives, however, were largely ineffective and only repressed luciferase activity to a maximum of 30% (Fig. 4C).

### Wnt antagonists are ineffective against liposomal Wnt

Ant1.4Br and Ant 1.4Cl compounds discriminate between purified Wnt3a, and Wnt3a secreted from cells in a paracrine/autocrine assay. This difference was not attributable to alterations in the protein itself either, since the paracrine Wnt signal was from L cells and these were the same cells used to produce the purified Wnt protein. Instead, these data imply that paracrine/autocrine signaling is mediated by a conformation of Wnt that differs from the purified protein. One possibility is that Wnt secreted by cells during autocrine/paracrine signaling is associated with a lipid raft or vesicle that facilitates its transport [21] and that liposomal packaging mimics this biological state.

We hypothesized that association of Wnt with a lipid vesicle, either during autocrine/paracrine signaling or when packaged in a liposome, might impede the activity of the antagonists. We directly tested this hypothesis by treating LSL SuperTOPflash cells with purified Wnt3a or liposomal Wnt3a in the presence of Ant 1.4Cl. Ant1.4Cl blocked ∼80% of exogenous Wnt3a-induced luciferase activity, which was comparable to the inhibitory effect of hFzd8-CRD (Fig. 4D). When we treated SuperTOPflash cells with liposomal Wnt3a and Ant1.4Cl, we observed a significant reduction in inhibitory potential of the antagonist. Again, hFzd8-CRD was effective in blocking liposomal Wnt3a-induced luciferase activity (Fig. 4D). Thus, Ant 1.4Br and Ant 1.4Cl were potent antagonists of purified Wnt3a under a variety of assay conditions but were ineffective against autocrine, paracrine, and liposomal Wnt3a.

### Liposomal packaging of Wnt3a potentiates its biological activity in vivo

Secreted Wnt is different than purified Wnt, perhaps because the former is associated with a lipid raft. Some data suggest that Wnts are associated with a lipid membrane when they are shuttled between cells [21]–[23]. If this hypothesis is true then we reasoned that the *in vivo* efficacy of the purified protein would be enhanced by packaging Wnts in liposomes.

**Figure 5:**
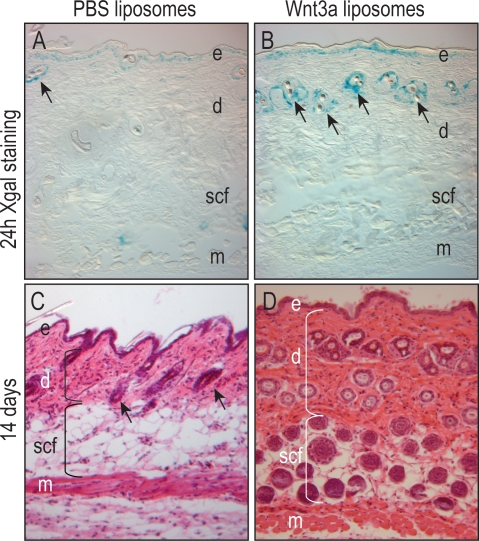
Wnt3a liposomes have *in vivo* activity. (A) Equal volumes of PBS or Wnt3a liposomes were injected subcutaneously into Axin2LacZ/+ reporter mice. Xgal staining at 24 h revealed reporter activity in cells at the epidermal/dermal junction and surrounding hair follicles (arrows). (B) Treatment with Wnt3a liposomes resulted in stronger Xgal staining with no change in the distribution of reporter activity. Over the intervening time period, both groups received injections of PBS liposomes or Wnt3a liposomes every other day. On day 14, tissues were collected. (C) Repeated injection of PBS liposomes had no effect on dermal thickness or hair follicle number (n = 6). (D) In contrast, repeated injections of Wnt3a liposomes resulted in dermal thickening and a dramatic increase in the number of hair follicles at the site of injection (n = 6). e, epidermis; d, dermis; scf, subcutaneous fat; m, muscle.

We tested the efficacy of the Wnt3a liposomes in an *in vivo* context, where genetic experiments have demonstrated a function for β-catenin dependent Wnt signaling in hair follicle neogenesis [24]. Wnt3a liposomes or PBS (control) liposomes were injected subcutaneously into the dorsal surface of 6 week old Axin2LacZ/+ mice, in which the LacZ gene is under control of the endogenous Wnt target Axin2 [25], [26]. Twenty-four hours later, Xgal staining was considerably stronger in the epidermal/dermal junction and around hair follicles in animals treated with Wnt3a liposomes compared to those treated with PBS liposomes (n = 6 for each condition; Fig. 5A,B). The cellular distribution of the staining, however, was unchanged (Fig. 5A,B).

We assessed the tissue response to Wnt3a liposomes. Sites treated with PBS liposomes showed no discernable change from un-injected controls on post-injection d14 (n = 6; Fig. 5C). In sharp contrast, sites treated with Wnt3a liposomes exhibited robust hair follicle neogenesis (n = 6; Fig. 5D). The dermal thickness was increased by 2-fold and there were significantly more hair follicles within that layer; in addition, the region normally containing subcutaneous fat was filled with hair follicles (Fig. 5D).

## Discussion

### Liposomal packaging potentiates the effects of Wnt proteins in vivo

Because of their potential therapeutic value, multiple large scale screens have been conducted in an attempt to identify small molecule agonists of the Wnt pathway [4]. Although molecules that synergize with Wnt proteins have been uncovered, none act as agonists in the absence of Wnts. This characteristic appears to be unique to Wnts. Unlike other pathways regulated by lipid-modified morphogens including Hedgehogs, Wnt signaling requires the assembly of two receptors plus a large and complex cytoplasmic group of molecules including Axin, Dsh, and GSK-3β. This may indeed only by accomplished by Wnt protein in the proper configuration and not by other molecules. Furthermore, most assays and experiments rely on conditioned media or viral over-expression approaches to study the effects of Wnts on cell function, which oftentimes confounds the interpretation of experimental results because of the inherent inability to control the amount, duration, and activity of Wnts.

With the advent of methods to purify Wnt proteins [1], [2] some of these difficulties have been circumvented but Wnt proteins are not stable for extended periods of time (Fig. 2). We found that liposomal packaging enhanced and sustained the biological activity of Wnt proteins both *in vitro* and *in vivo* (Fig. 2). Furthermore, liposomal Wnt3a exhibited the same biological specificity as purified Wnt3a, as demonstrated by the patterns of Xgal staining following delivery in multiple strains of Wnt reporter mice (Fig. 5 and data not shown).

### Liposomal packaging mimics biological transport

Wnt proteins contain a lipid adduct that is required for activity ([1]; reviewed in [27], [28]) but precisely how this lipid modification affects the intercellular transport of Wnt proteins is unclear. Some evidence suggests that palmitoylation is required for Wnt secretion [29], [30]. The lipid adduct may be the method by which Wnt is tethered to a cell membrane, which in turn would restrict diffusion and thus allow local concentration of Wnt proteins to reach a threshold level required for biological activity [31].

### A biomimetic approach for Wnt delivery in vivo

In *Drosophila* there is some evidence suggesting that the Wg protein is transported over many cell diameters in small vesicular structures [22]. These data hint at an appealing hypothesis: liposomal packaging mimics the method by which Wnts are normally secreted from cells. Four lines of experimental evidence support this conclusion. First, liposomal packaging potentiates the activity of purified Wnt3a. Second, Wnt antagonists that are effective against purified protein and are ineffective against Wnt secreted from cells are also ineffective against liposomal Wnt. This selective antagonism is likely due to interactions between the antagonists and a region of the Wnt protein that is available in its purified state, but hidden when Wnt is secreted in a paracrine manner or if Wnt is associated with a liposome. Third, liposomal Wnt3a increases reporter activity *in vivo* but does not alter the distribution of Wnt responsive cells. These data suggest that liposomal Wnt3a acts similar to endogenous Wnts. Fourth, Wnt3a liposomes exhibit robust activity in a biologically relevant model of hair follicle neogenesis.

### Therapeutic strategies to exploit liposomal Wnts

Given the role of Wnt signaling in many regenerative processes (planaria; fish tails) the delivery of Wnt protein as a biological reagent has obvious clinical applications. In addition to its well described role in inducing hair development and growth [24], [32], Wnts are also implicated in the self-renewal and proliferation of hematopoietic stem cells [2]; mesenchymal stem cells; and neural stem cells. Wnts may also be an effective means to stimulate bone formation after injury or in disease states such as osteoporosis [33]. Any strategy that attempts to target the Wnt pathway to augment tissue regeneration will have to take into consideration the need to selectively and locally activate signaling in the tissue or area of interest, whilst simultaneously restricting Wnt signaling in other parts of the body. Future experiments will focus on the potential to stimulate tissue regeneration using Wnt liposome based approaches.

## Supporting Information

Figure S1 Liposomal packaging does not impair Wnt activity. (A) An in vitro Wnt3a activity gradient was generated by measuring activity with increasing Wnt3a concentrations in the media. (B) Different volumes of liposomal Wnt3a and purified Wnt3a were added to LSL cells; the activity of liposomal Wnt3a corresponded to an effective Wnt3a concentration of 1.0 µg/ml. (n = 3; mean+standard deviation).
(2.51 MB TIF)
