# Supplementary figures and images for: Liposomal Packaging Generates Wnt Protein with In Vivo Biological Activity
Source: PLoS One. 2008 Aug 13;3(8):e2930. doi: 10.1371/journal.pone.0002930 (PMC2515347; doi:10.1371/journal.pone.0002930)

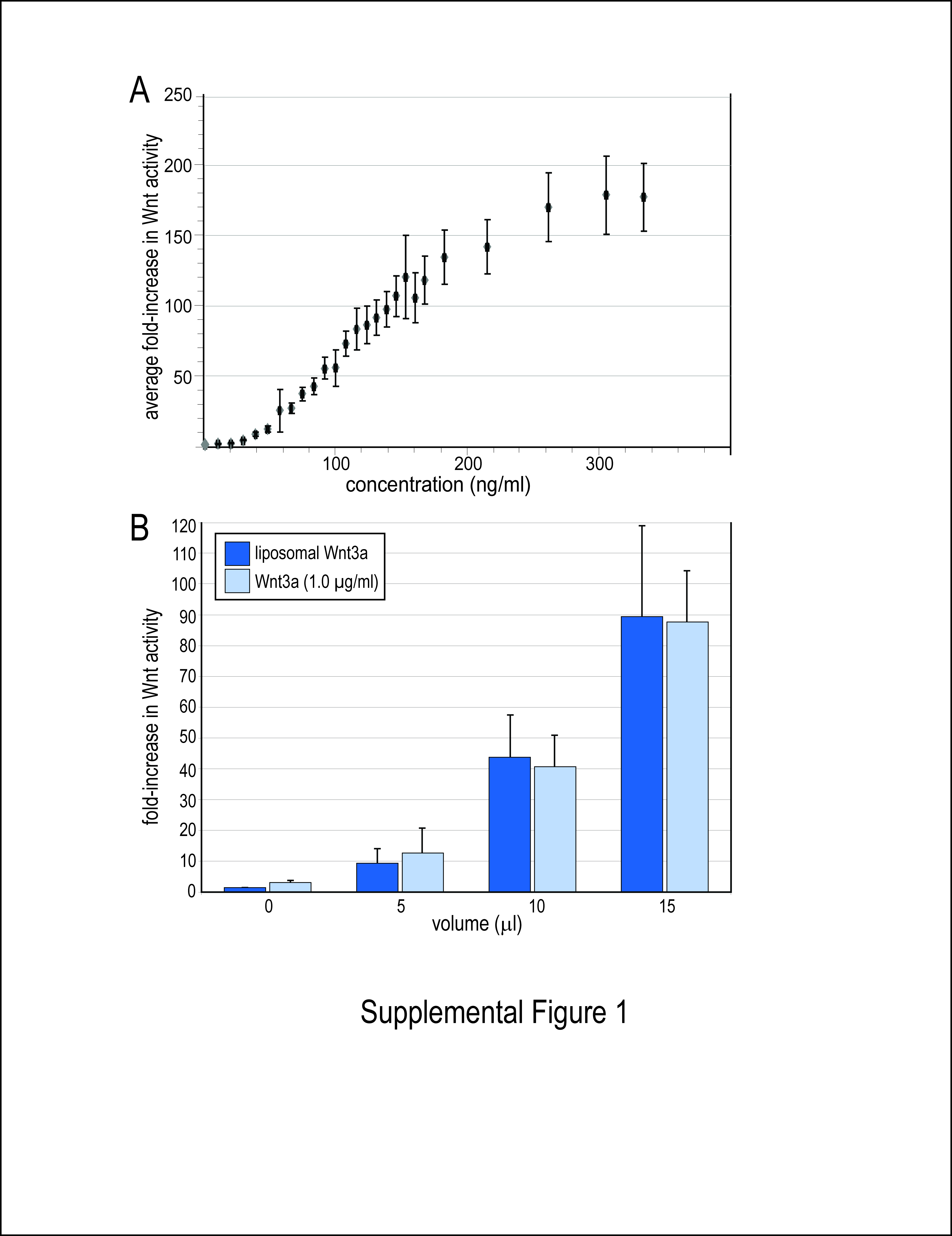

Supplement: Figure S1 — Liposomal packaging does not impair Wnt activity. (A) An in vitro Wnt3a activity gradient was generated by measuring activity with increasing Wnt3a concentrations in the media. (B) Different volumes of liposomal Wnt3a and purified Wnt3a were added to LSL cells; the activity of liposomal Wnt3a corresponded to an effective Wnt3a concentration of 1.0 µg/ml. (n = 3; mean+standard deviation). (2.51 MB TIF) [file pone.0002930.s001.tif]
